# Artery of Percheron Infarction: Diagnostic and Clinical Insights From Two Cases

**DOI:** 10.7759/cureus.95907

**Published:** 2025-11-01

**Authors:** Ashlin Z Thomas, Ashtami Krishnan, Hannah Joseph, Arjun Sreekumar, Vishnu Jayachandran, Philip Mathew

**Affiliations:** 1 Department of Critical Care Medicine, Believers Church Medical College Hospital, Thiruvalla, IND; 2 Department of Neurology, Believers Church Medical College Hospital, Thiruvalla, IND; 3 Department of Cardiothoracic Surgery, Dr. K M Cherian Institute of Medical Sciences, Kallissery, IND

**Keywords:** altered sensorium, artery of percheron, bilateral paramedian thalamic infarct, mri, rostral midbrain stroke

## Abstract

Artery of Percheron (AOP) infarction is a rare cause of bilateral paramedian thalamic and rostral midbrain stroke, typically presenting with altered consciousness, vertical gaze palsy, memory deficits, and other nonspecific neurological symptoms. Diagnosis is often delayed due to its variable clinical presentation and the limited sensitivity of initial CT imaging. We report two cases of AOP infarction confirmed by magnetic resonance imaging (MRI), both presenting with reduced responsiveness and requiring intensive care management. MRI revealed bilateral paramedian thalamic infarcts consistent with AOP territory involvement. These cases emphasize the need for early clinical suspicion and prompt MRI evaluation in patients with unexplained altered sensorium.

## Introduction

The thalamus is a diencephalic nuclear complex that plays a key role in memory, emotions, sleep-wake cycle regulation, executive processes, cortical alertness, and sensorimotor integration [1]. The lateral, medial, and posterior thalamic regions are primarily supplied by the vertebrobasilar system through branches of the posterior cerebral and posterior communicating arteries [2].

The artery of Percheron (AOP) is an uncommon anatomical variant in which a single arterial trunk arises from the P1 segment of one posterior cerebral artery and supplies both paramedian thalami and the rostral midbrain [3]. Occlusion of the AOP accounts for approximately 0.1%-2% of all ischemic strokes and 4%-18% of all thalamic strokes [4]. This can result in bilateral paramedian thalamic infarction, occasionally involving the rostral midbrain, leading to a wide spectrum of neurological manifestations.

Given the thalamus’s involvement in multiple cognitive and motor functions, AOP infarction often presents with diverse symptoms, including altered consciousness, supranuclear vertical gaze palsy, memory impairment, aphasia, somnolence, sensory or motor deficits, confusion, stupor, or even coma [4,5]. The nonspecific nature of these clinical features frequently delays diagnosis and treatment.

We report two patients presenting with reduced responsiveness who were found to have AOP infarction on MRI, highlighting the diagnostic challenge associated with this uncommon condition and underscoring the importance of considering it among the differential diagnoses of bilateral thalamic syndromes.

## Case presentation

Case 1

A 51-year-old woman with no known comorbidities presented with complaints of dysarthria and decreased responsiveness. She was initially evaluated at an external facility, where she was intubated due to a decline in her level of consciousness (Glasgow Coma Scale (GCS): E2V2M3).

On initial examination, she was unresponsive with sluggish bilateral pupillary reactions. Further neurological assessment could not be performed due to the prior administration of muscle relaxants during intubation. Despite these challenging circumstances limiting detailed evaluation, no metabolic abnormalities were identified to account for her symptoms, suggesting a potential intracranial cause.

**Figure 1:**
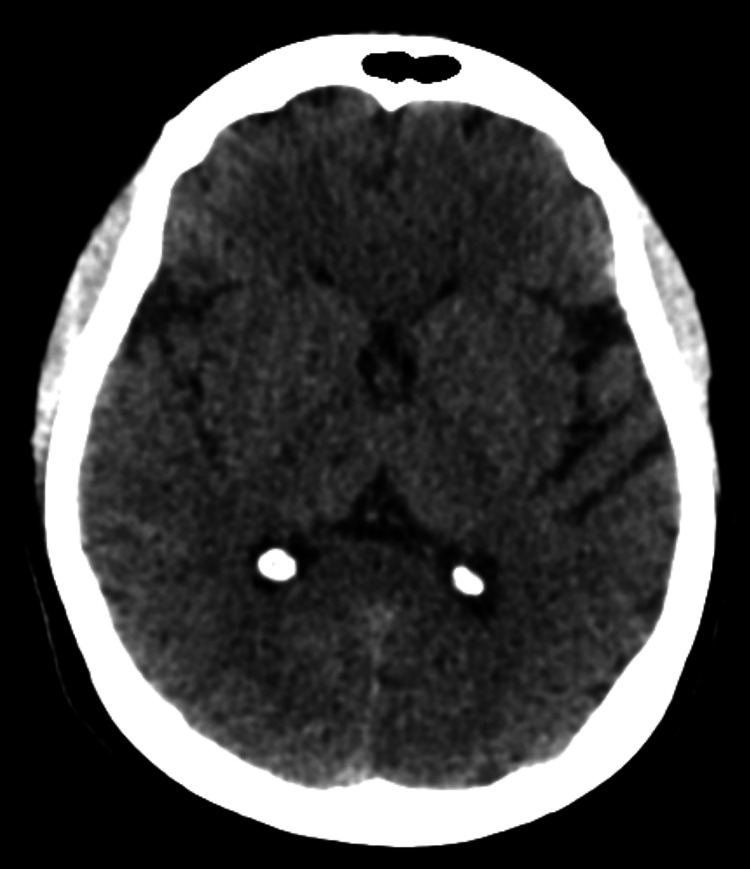
CT head without contrast does not show any thalamic infarct.

The initial diagnostic step, a CT brain, showed no abnormalities, which prompted further evaluation with MRI (Figure 1).

**Figure 2:**
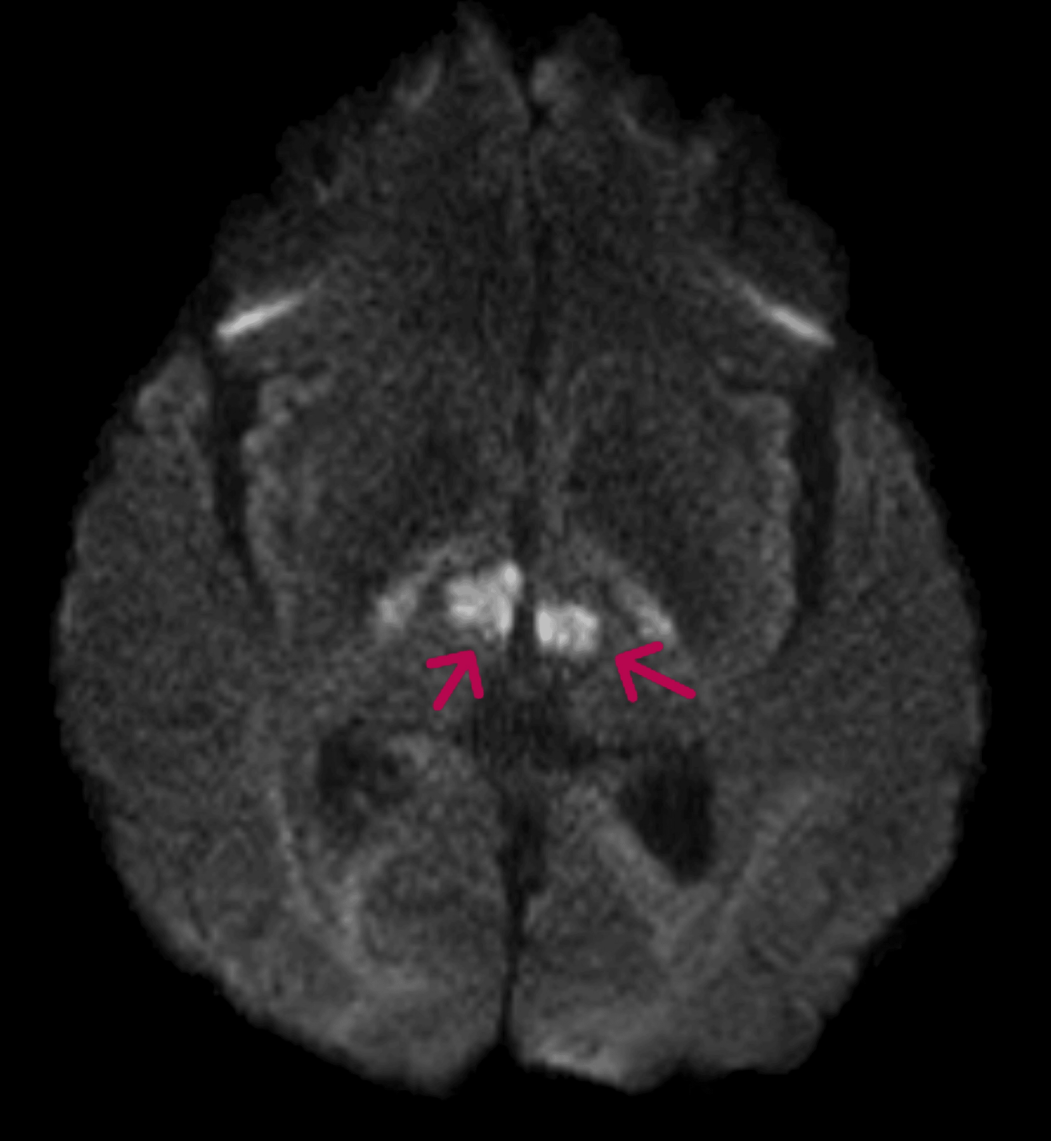
MRI brain (DWI sequence) showing diffusion hyperintensity in the bilateral thalami (arrows). DWI: Diffusion-weighted imaging

**Figure 3:**
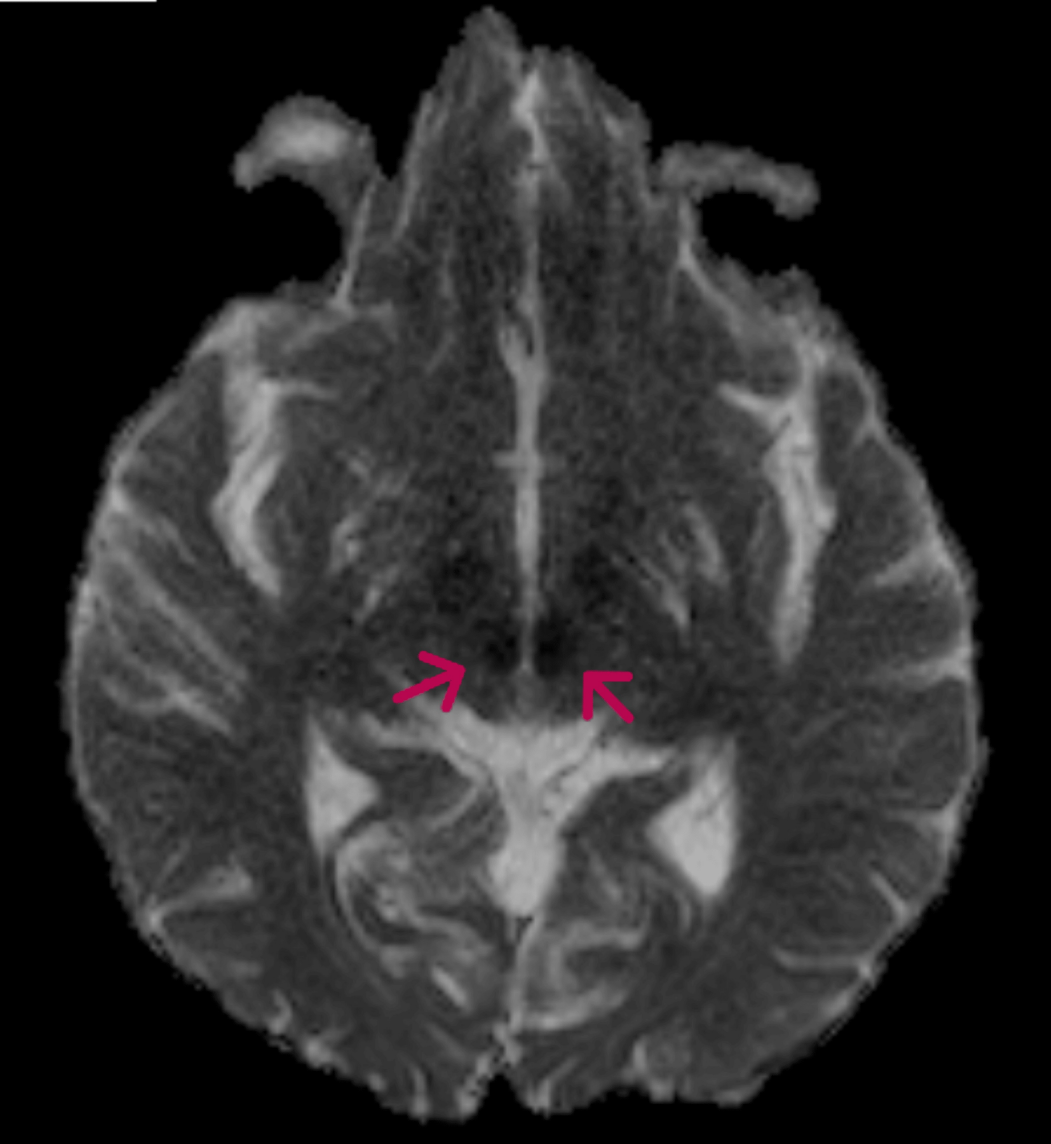
MRI brain (ADC sequence) showing fall in ADC mapping in the bilateral thalami (arrows). ADC: Apparent diffusion coefficient

**Figure 4:**
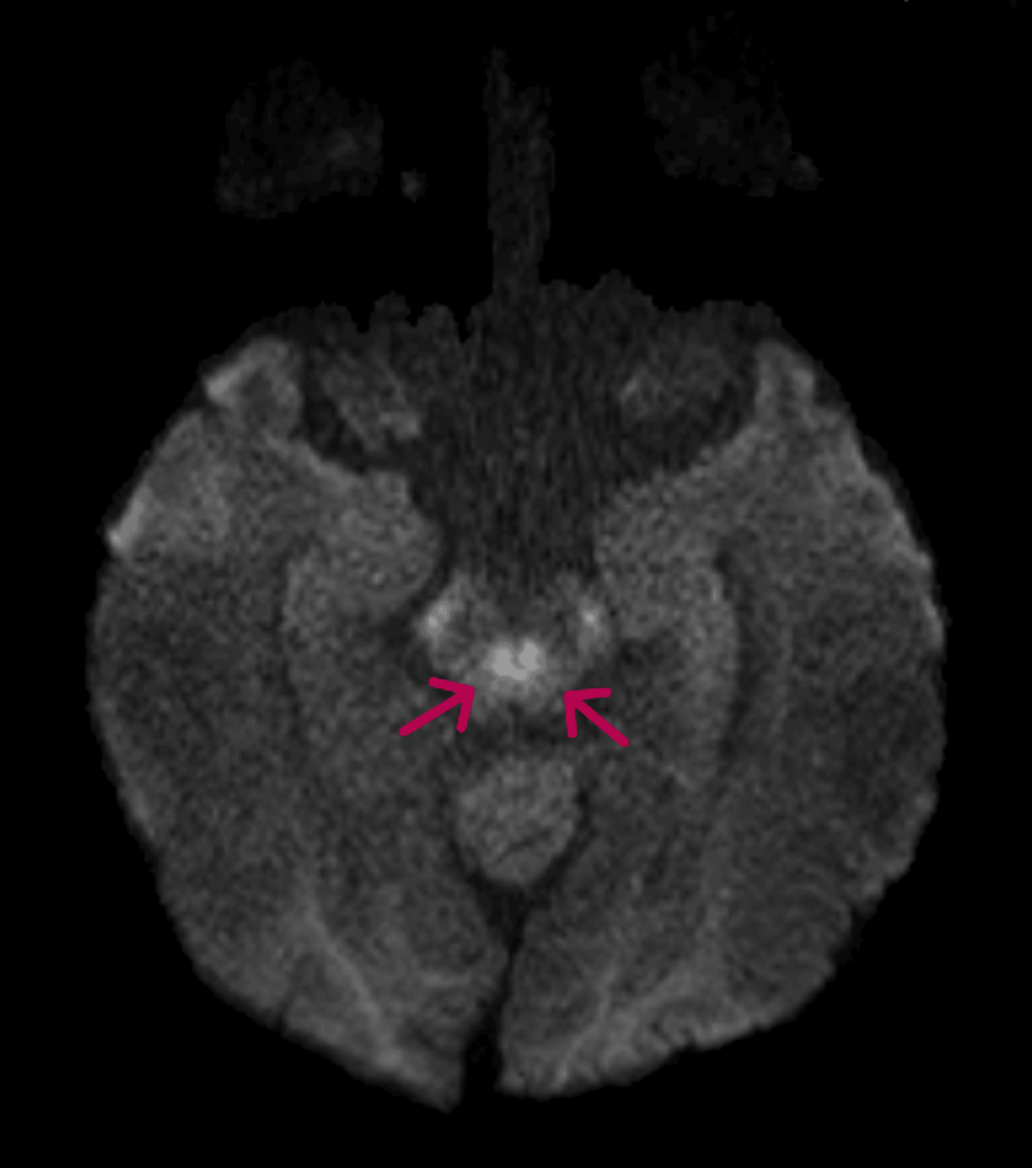
MRI brain (DWI sequence) shows diffusion hyperintensity in rostral midbrain (arrows). DWI: Diffusion-weighted imaging

The MRI demonstrated diffusion restriction on DWI involving the bilateral paramedian thalami and rostral midbrain (left greater than right), consistent with an acute infarct (Figures 2-4).

As the patient presented about six hours post-onset of symptoms, she was beyond the intravenous thrombolysis window of ≤4.5 hours. Her NIH Stroke Scale (NIHSS) was estimated to be 14, consistent with a moderate stroke. Risk of hemorrhagic transformation was considered to be low based on her radiological findings and normal coagulation screen. Based on clinical acumen and presentation outside the thrombolytic window, she was initiated on dual antiplatelet therapy, a statin, and other supportive measures.

Subsequent neurological examination revealed central ptosis, memory disturbances, and hypersomnolence. She also demonstrated right-sided hemiparesis (power 3/5). Over the following days, her GCS gradually improved, and she was successfully extubated after 20 days. Her modified Rankin Scale (mRS) was 4 at 30 days post-onset of symptoms. Despite residual hemiparesis (power 4/5) and persistent hypersomnolence, she made a steady recovery, as reflected by improvement in the NIHSS from 14 on admission to 8 on discharge. She was ultimately discharged after two months with improvement in her attentiveness and alertness. 

Case 2

A 68-year-old woman with a history of type 2 diabetes mellitus and coronary artery disease was found with decreased responsiveness and aphasia at home and promptly transported to a local hospital. On admission, she was noted to be in atrial fibrillation (AF), despite having no prior history of arrhythmia. She was intubated due to a GCS of 7 (E2V2M3), accompanied by hypercapnia and respiratory acidosis.

Upon transfer to our tertiary care center, the patient remained drowsy with minimal response to external stimuli. Bilateral plantar extensor responses were observed, and pupils were sluggishly reactive to light. Central ptosis was also noted. No neck stiffness or other cranial nerve deficits were identified. Assessment of memory and orientation was limited due to her reduced responsiveness.

**Figure 5:**
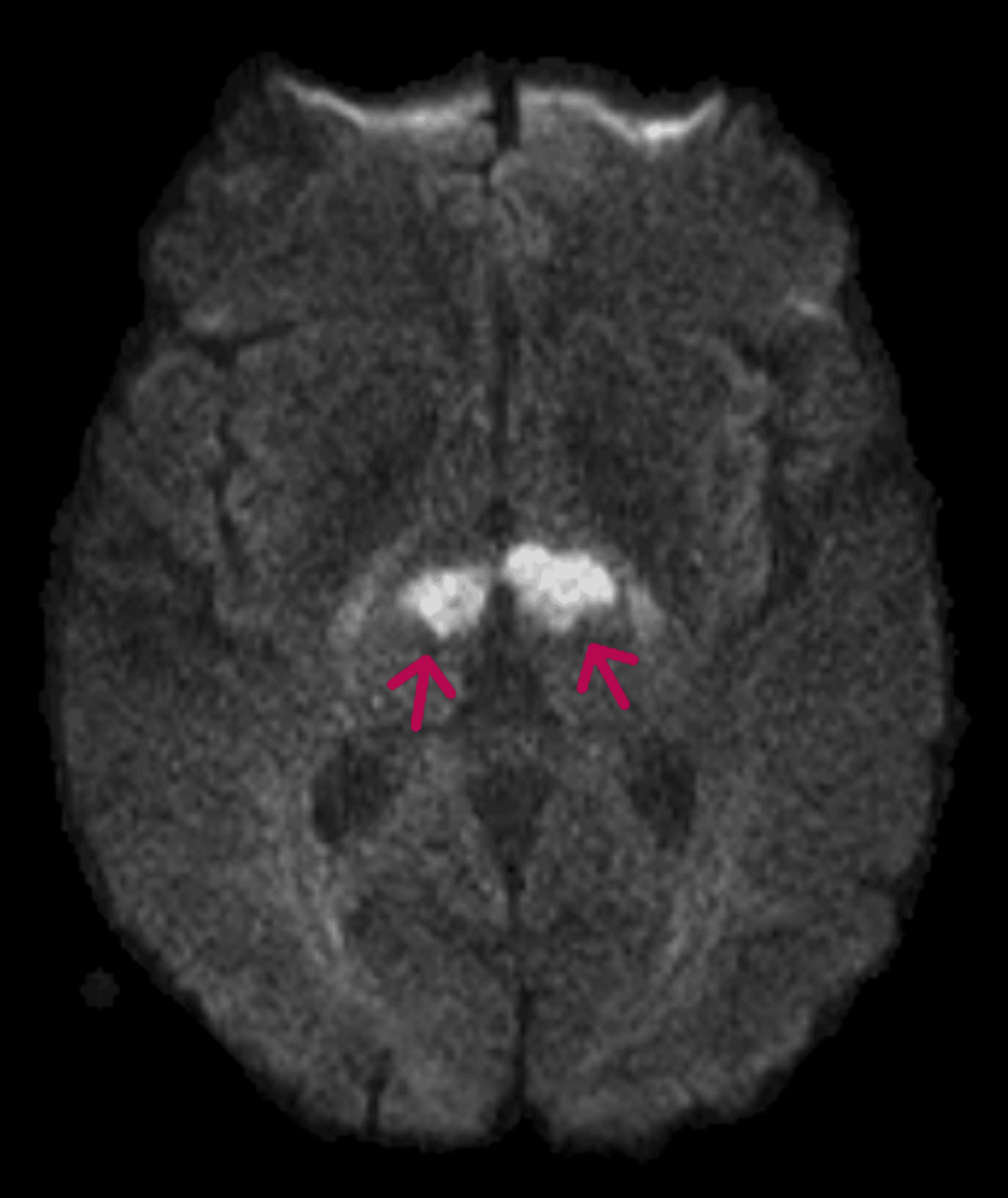
MRI brain (DWI sequence) showing diffusion hyperintensity in the bilateral thalami (arrows). DWI: Diffusion-weighted imaging

**Figure 6:**
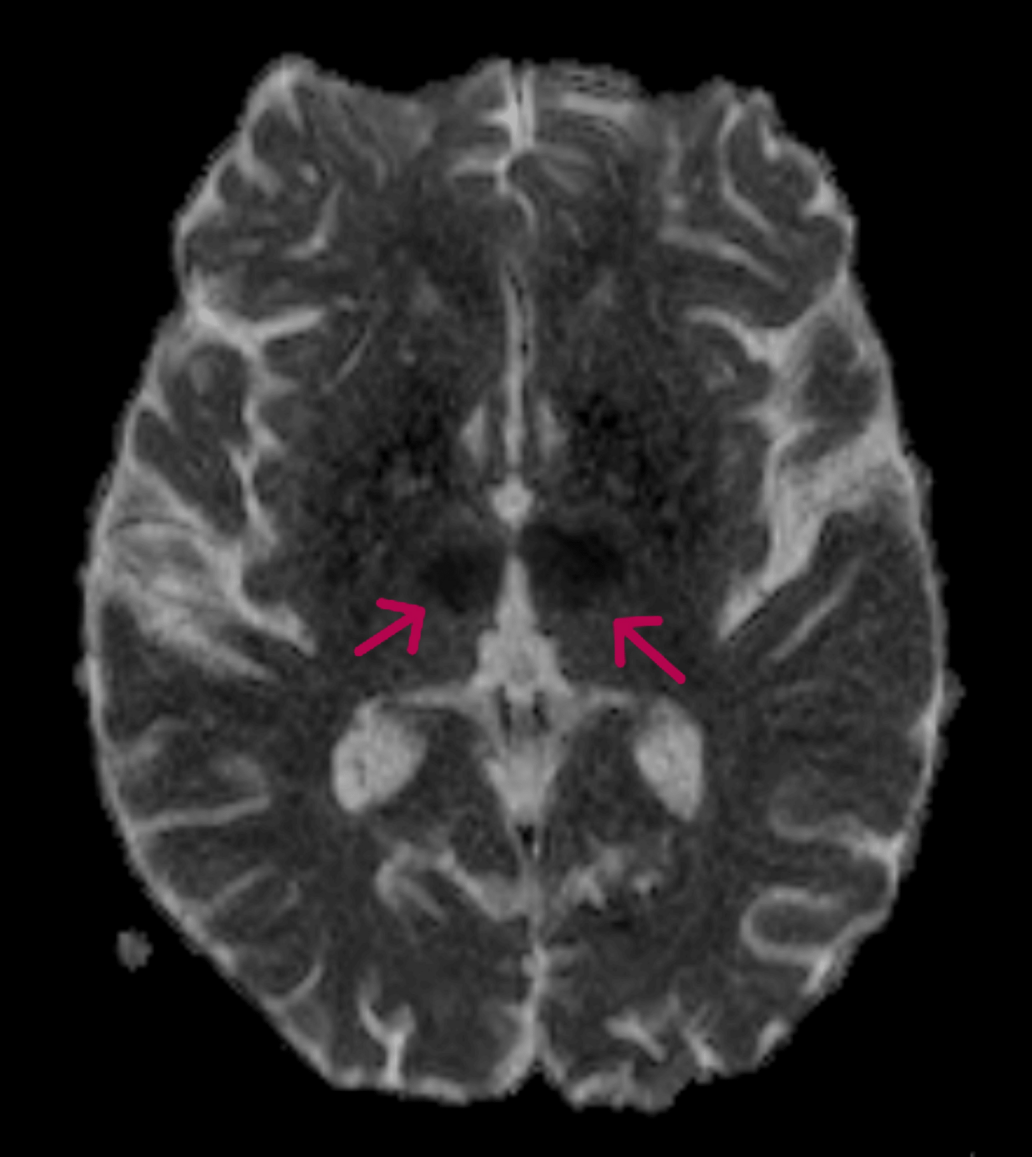
MRI brain (ADC sequence) showing a fall in ADC mapping in the bilateral thalami (arrows). ADC: Apparent diffusion coefficient

An initial non-contrast CT scan performed at the referring hospital was normal, showing no intracranial hemorrhage. Given her new-onset atrial fibrillation and high embolic risk, an MRI of the brain was obtained approximately six hours after the onset of symptoms. The scan demonstrated acute diffusion restriction involving the bilateral anteromedial thalami, consistent with an infarct in the AOP territory (Figures 5, 6). 

Magnetic resonance angiography (MRA) of the posterior circulation showed patent basilar and posterior cerebral arteries, with no evidence of large-vessel occlusion. Although the single AOP trunk could not be visualized, the characteristic pattern of bilateral thalamic involvement supported the diagnosis.

As she presented at six hours post-onset of symptoms, beyond the window for intravenous thrombolysis of ≤4hours, with a higher NIHSS score of 18, she was managed with single antiplatelet therapy, a statin, and other supportive measures. A detailed cardiology evaluation, including Holter monitoring, confirmed paroxysmal AF. Oral anticoagulation with apixaban was initiated on day 14 of hospitalization after a non-contrast CT scan confirmed the absence of hemorrhagic transformation. Before initiating anticoagulation, the bleeding risk was assessed using the HAS-BLED score and calculated to be 2.

The severity of her condition necessitated a tracheostomy for prolonged mechanical ventilation. Her hospital course was further complicated by ventilator-associated pneumonia and recurrent episodes of AF, which were managed with targeted antibiotic therapy and cardioversion.

After nearly three weeks of intensive care and physiotherapy, the patient’s sensorium gradually improved. Despite a prolonged hospital stay, which included tracheostomy care and oxygen support (discontinued after two months), she was discharged after three months with the tracheostomy tube in place; it was subsequently removed during follow-up. Her consistent adherence to rehabilitation and treatment facilitated significant progress throughout her hospitalization. The NIHSS score improved from 18 on admission to 9 at discharge. The mRS was 4 at 30 days and 2 at 90 days, reflecting substantial functional improvement over her three-month stay. At discharge, she had regained mobility and near independence, highlighting the combined impact of structured medical care and patient resilience on her recovery.

## Discussion

The thalamus comprises multiple nuclear groups, including the anterior, medial, lateral, ventral, and intralaminar nuclei. These nuclei communicate with various regions of the brain and mediate numerous functions, such as arousal, alertness, subcortical perception, memory, emotions, sleep integration, language comprehension, and sensory relay [6]. The thalamus is supplied with blood from four primary vascular regions: the tuberothalamic, inferolateral, paramedian, and posterior choroidal arteries. The paramedian artery is mainly responsible for supplying the dorsomedial nucleus, internal medullary lamina, and intralaminar nuclei [7]

**Figure 7:**
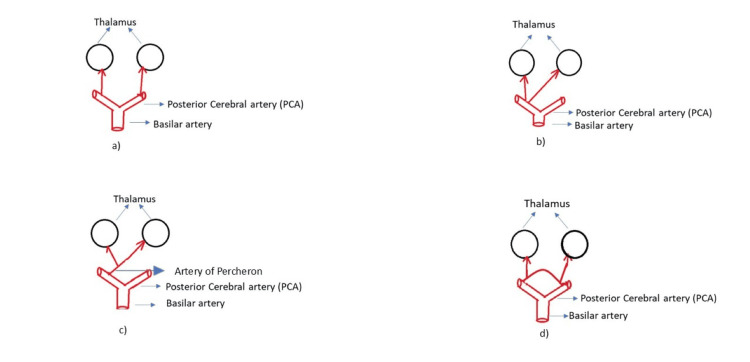
Variants of the paramedian thalamic arteries. a: Type I – Normal variant, with each paramedian artery originating from the P1 segment of the posterior cerebral artery (PCA) on the corresponding side. b: Type IIa – Both paramedian arteries originate from either the left or right P1 segment. c: Type IIb – Artery of Percheron (AOP) variant, arising from one P1 segment and bifurcating to supply the bilateral paramedian thalami and rostral midbrain; this is the variant involved in our patient’s infarction. d: Type III – An arterial arcade connects the right and left P1 segments, giving rise to bilateral paramedian arteries. Illustration created by the authors based on the description of the four variants of the paramedian artery by Gerard Percheron in [8]

In 1973, Gerard Percheron described four variants of the paramedian artery [8], which are illustrated in Figure 7.

The dorsomedial nucleus directs attention to important sensory stimuli and is involved in processing emotions through connections with the prefrontal cortex and hypothalamic nuclei. The intralaminar nuclei contribute to attention, orientation, memory processing, and reward-based behavior [9].

In the first patient, MRI revealed infarction involving both paramedian thalami and the rostral midbrain, left side greater than the right. Altered consciousness and hypersomnolence may reflect involvement of the paramedian thalamic nuclei and disruption of the ascending reticular activating system (ARAS). Memory impairment is consistent with involvement of the dorsomedial nuclei and their connections with the prefrontal cortex and limbic system. Central ptosis may correspond to ischemic involvement of the oculomotor nuclear complex or its fascicular fibers within the rostral midbrain. Right-sided hemiparesis may be related to partial involvement of corticospinal tract fibers in the midbrain tegmentum, corresponding to the left-sided predominance of the lesion on MRI.

In contrast, the second patient had MRI findings confined to the bilateral anteromedial thalami without midbrain involvement. Decreased responsiveness may be due to disruption of ARAS projections within the medial thalamic nuclei. Transient aphasia may reflect interruption of thalamocortical connections between the dominant thalamus and cortical language areas. Mild ptosis, despite no visible midbrain lesion, may represent secondary edema or microinfarction affecting periaqueductal gray matter supplied by the same vascular territory. The absence of hemiparesis is consistent with sparing of corticospinal tracts. These differences illustrate how variable extension of AOP infarction may contribute to the diversity of clinical presentations.

Both patients initially presented with reduced responsiveness. Dysarthria was prominent in the first patient, while the second patient presented with aphasia, which gradually improved during hospitalization. Both exhibited fluctuating alertness and somnolence, likely reflecting involvement of paramedian thalamic nuclei and ARAS projections.

Initial evaluation in the Emergency Department requires detailed history, careful examination, and timely selection of the appropriate imaging modality. CT head without contrast was performed first, but it did not localize acute changes. Given the high suspicion of stroke, an MRI brain stroke protocol was performed. Diffusion restriction involving the bilateral paramedian thalami and rostral midbrain was observed in the first patient, supporting AOP territory involvement. This underscores the importance of early detection, particularly when patients present within the window for intravenous thrombolysis, as timely administration of tissue plasminogen activator is critical.

In suspected AOP infarction, the small-caliber artery is often not visualized on CT or MRA. Non-contrast CT was first performed to rapidly exclude intracranial hemorrhage. CTA was not performed, as both patients presented beyond the window for thrombolysis. Definitive visualization of bilateral paramedian thalamic infarcts was achieved using MRI with diffusion-weighted imaging. MRA of the posterior circulation confirmed patency of the basilar and posterior cerebral arteries, allowing identification of a small vessel infarct such as AOP occlusion in a timely manner.

## Conclusions

AOP infarction, though rare, poses a diagnostic challenge due to its variable clinical manifestations and subtle imaging findings. These cases underscore the importance of considering AOP infarction in patients presenting with altered consciousness, dysarthria, central ptosis, or memory deficits, even when other neurological signs are subtle. Advanced imaging, particularly MRI with diffusion-weighted sequences, is essential for accurate diagnosis. Early recognition, timely intervention, and a structured multidisciplinary approach can improve outcomes, facilitating meaningful recovery even in patients with severe initial presentations. Awareness of this condition is crucial to avoid misdiagnosis and ensure optimal patient care.
